# A changing early onset scoliosis phenotype in spinal muscular atrophy: nusinersen treatment is associated with younger age, increased curve magnitude, and kyphosis at the time of index surgery

**DOI:** 10.1007/s43390-026-01359-1

**Published:** 2026-09-17

**Authors:** Ian Hollyer, Talissa Generoso, Bill Young, Xochitl Bryson, Nicole Segovia, Marleni Albarran, Majella Vaughan, Amy Mcintosh, Brandon Ramo, Daniel Bouton, Brian Snyder, Michael Vitale, John Smith, Benjamin Roye, Patrick Cahill, Jaysson Brooks, Ron El-Hawary, Pediatric Spine Study Group, John S Vorhies

**Affiliations:** 1 Department of Orthopaedic Surgery, Stanford University School of Medicine, 453 Quarry Rd, 3rd Floor, Palo Alto, CA MC 5658 USA; 2 Pediatric Spine Foundation, Valley Forge, PA USA; 3 Scottish Rite for Children, Dallas, TX USA; 4 Kaiser Permanente of Northern California, Oakland, CA USA; 5 Boston Children’s Hospital, Boston, MA USA; 6 Columbia University Medical Center, New York City, NY USA; 7 University of Utah Health, Salt Lake City, UT USA; 8 Children’s Hospital of Philadelphia, Philadelphia, PA USA

**Keywords:** Early onset scoliosis, Spinal muscular atrophy, Nusinersen

## Abstract

**Background:**

Nusinersen provides functional benefits to Spinal Muscular Atrophy (SMA) patients but does not affect the prevalence or progression of scoliosis. This study aims to characterize perioperative curve characteristics and patient demographics among patients with SMA undergoing surgical treatment for early onset scoliosis (EOS) in a period spanning the introduction of nusinersen, a disease-modifying therapy.

**Methods:**

This retrospective cohort study utilized a multicenter international pediatric spinal deformity database. Patients with SMA undergoing initial surgical treatment for scoliosis between 1996 and 2023 were included. Patients were stratified by exposure to nusinersen preoperatively. Preoperative clinical, radiographic characteristics, and first erect postoperative radiographic parameters were compared between groups.

**Results:**

A total of 210 patients were included, 37 with preoperative nusinersen exposure and 173 controls. Patients in the nusinersen group were younger at the time of intervention (6.5 vs. 7.6 years, *p* = 0.006) and had more spinal levels instrumented post-operatively (10.5 vs. 9.5, *p* = 0.033). Preoperative kyphosis (78° vs. 67°, *p* = 0.027) and major curve magnitude (83° vs. 75°, *p* = 0.030) were higher in the nusinersen group. Postoperative kyphosis was also greater in this group (48° vs 42°, *p* = 0.042). There was no difference in postoperative major curve magnitude or percent correction. The nusinersen group was more commonly treated with magnetically controlled growing rods. A contemporaneous subgroup analysis (post-2017) showed that patients treated with nusinersen were younger at the time of surgery, although no significant differences in preoperative curve characteristics were noted.

**Conclusion:**

Nusinersen exposure was associated with younger age at surgery, greater curve magnitude, and increased kyphosis. These findings highlight potential changes in the phenotype of surgical candidates with EOS and SMA among patients treated with Nusinersen.

**Supplementary Information:**

The online version contains supplementary material available at https://doi.org/10.1007/s43390-026-01359-1.

## Introduction

Spinal muscular atrophy (SMA) is an autosomal recessive progressive neuromuscular disorder consisting of a mutation in the survival motor neuron 1 gene (SMN1) that results in gradual deterioration of motor neurons [1, 2]. One of the most common genetic disorders, SMA has an estimated incidence of around 1 in every 3,900 to 16,000 births annually and leads to severe proximal muscle weakness and atrophy [3].

The clinical progression of SMA and associated lower motor neuron dysfunction includes hypotonia, fasciculations, hyporeflexia, muscle atrophy, and muscle weakness (including decreased pulmonary function) [4]. SMA is traditionally classified into three types based on age of onset and classical phenotypic descriptions of clinical severity, where type I patients (onset < 6 months) cannot sit independently, type II patients (onset 6–18 months) can sit but not ambulate, and type III patients (onset > 18 months) can ambulate [5]. In more severely affected patients, muscle weakness can lead to the development of scoliosis and abnormal pelvic obliquity, which can impact sitting balance and quality of life for patients [6, 7]. Scoliosis progression likely exacerbates deteriorating pulmonary function in these patients, which is already compromised given baseline muscular weakness [6]. Indications and thresholds have changed over the years but in general, progressive curves > 50 degrees are often treated surgically in patients with SMA with growth-friendly instrumentation in younger children and posterior spinal fusion in older individuals [8, 9].

Since the introduction in 2016 of nusinersen, a new class of disease-modifying agents (DMA) have emerged as treatment options for patients with SMA [10]. Nusinersen, an antisense oligonucleotide, was the first developed [11]. Nusinersen (Spinraza) modulates the expression of SMN2, a paralog gene of SMN1 that encodes a similar but more unstable protein product to SMN1 that can partially compensate for SMN1 deficiency, thereby facilitating production of functional SMN protein [12, 13]. Nusinersen has demonstrated functional benefits for motor function, muscle strength, range of motion and longevity [14–23]. A retrospective study of 165 patients with SMA found that DMA use was associated with a decreased risk of scoliosis development in a multivariate model, however the ability for DMA to prevent scoliosis is controversial and it is still unclear how nusinersen affects spinal curve characteristics in patients with SMA [7, 24]. Contrary to this study, it has been the authors’ observation that there is an increasing volume of SMA patients on nusinersen presenting for treatment to the pediatric spine provider, often with severe, stiff deformities at a young age.

This study aims to explore associations with nusinersen usage and perioperative scoliosis curve characteristics in patients with SMA undergoing surgical treatment for early onset scoliosis (EOS). We hypothesized that nusinersen administration would be associated with changes in scoliosis characteristics and postoperative curve correction achieved among patients with SMA and EOS.

## Methods

**Fig. 1:**
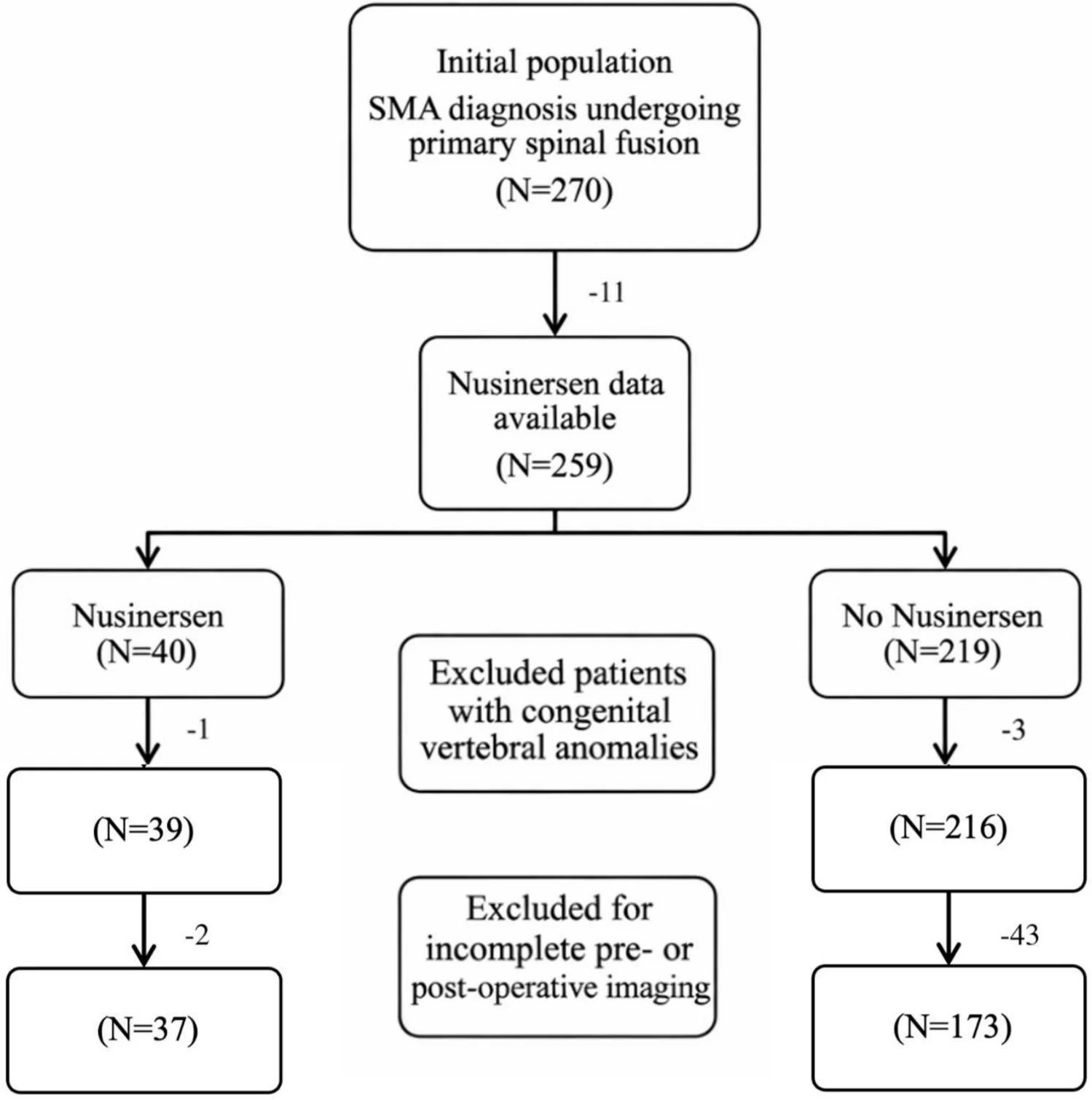
Flow chart of number of patients enrolled, including reasons for exclusion

This retrospective cohort study was conducted via a multicenter international pediatric spinal deformity database through the Pediatric Spine Study Group. Patients with SMA undergoing initial surgical treatment for scoliosis from 1996 through 2023 were identified. Inclusion criteria required patients to have upright preoperative coronal and lateral scoliosis radiographs as well as postoperative radiographs at any follow-up period. Exclusion criteria were patients with incomplete or unreadable imaging, patients with a history of previous spinal instrumentation or fusion (Fig. 1). Demographic data and surgical treatment details were collected from the patient's medical records. Radiographic parameters were collected from preoperative and postoperative imaging, including major coronal curve magnitude, the number of levels involved in the major coronal curve magnitude, the angle of maximum kyphosis, the levels involved in maximum kyphosis, and the pelvic obliquity (as measured by the Maloney method) [25].

Patients were stratified by preoperative exposure to nusinersen, with the control group comprised of patients with no exposure prior to surgical intervention. Preoperative clinical and radiographic characteristics and first postoperative radiographic parameters were compared between patients receiving nusinersen treatment and their control counterparts.

### Statistical analysis

Descriptive statistics were presented as means and standard deviations for continuous variables and as counts and percentages for categorical variables. Kruskal–Wallis, chi-square, and Fisher’s exact tests were used to evaluate demographic and clinical differences between patients treated with nusinersen and those not treated with nusinersen. These tests were repeated in three subanalyses looking at patients treated from 2017 onwards, patients treated with nusinersen for at least 1 year (compared to untreated patients), and patients treated with nusinersen for at least 2 years (compared to untreated patients). All analyses were completed in RStudio version 2023.03.1 + 446 (Boston, MA) using a two-sided significance level of 0.05.

## Results

**Fig. 2:**
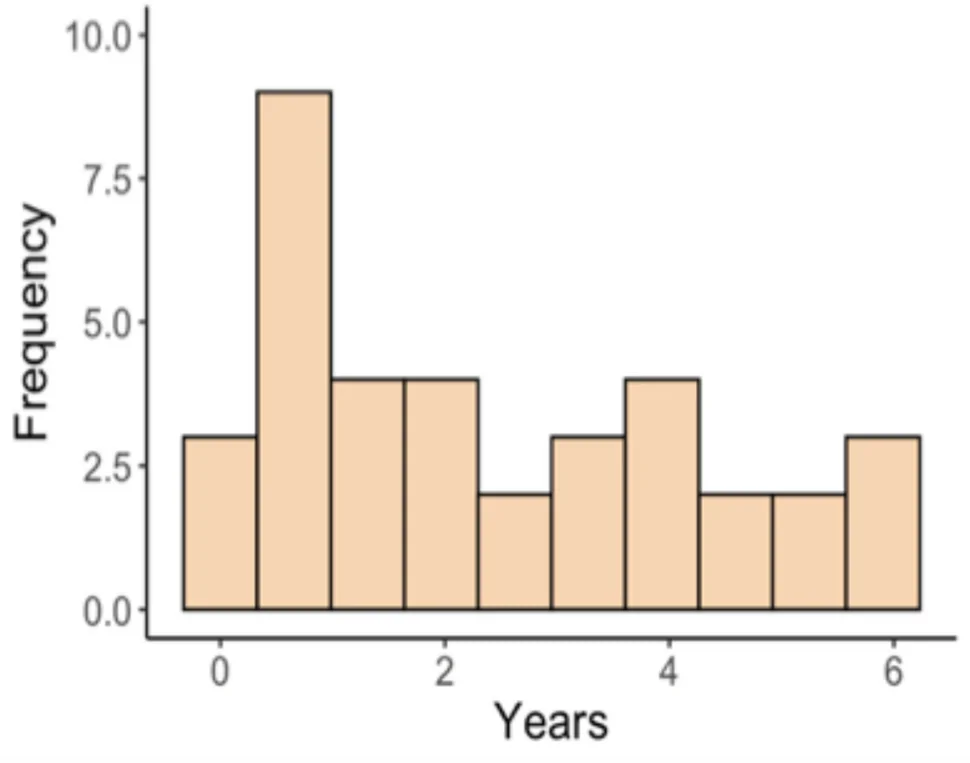
Histogram of total duration of treatment of nusinersen prior to surgery for each patient in the nusinersen group

A total of 210 patients were identified, 173 of whom were controls (never having been exposed to nusinersen prior to surgical treatment), and 37 had treatment with nusinersen preoperatively (Table 1). Patients in the nusinersen group had a median of 1.9 years (range: 0.02—5.9) of treatment with nusinersen before surgery (Fig. 2).

**Table 1:** Demographics, surgical, and curve characteristics by nusinersen use

	No nusinersen use (*n* = 173)		Nusinersen use (*n* = 37)		
	Mean (SD)	95% CI	Mean (SD)	95% CI	*p*-value
Age (years)	7.6 (2.8)	[7.2, 8.1]	6.5 (2.0)	[5.9, 7.2]	0.006*
BMI	16.8 (4.3)	[16.0, 17.5]	16.2 (3.0)	[14.9, 17.4]	0.389
Instrumented levels	9.5 (2.1)	[9.2, 9.9]	10.5 (2.5)	[9.7, 11.3]	0.033*
Pre-operative cobb angle	75.3 (21.9)	[72.0, 78.6]	83.3 (19.5)	[76.8, 89.8]	0.030*
Post-operative cobb angle	38.5 (16.1)	[36.1, 40.9]	45.3 (19.3)	[38.9, 51.7]	0.052
Percent change in cobb angle	− 47.9 (20.5)	[− 51.0, − 44.8]	− 46.3 (16.6)	[− 51.9, − 40.8]	0.617
Pre-operative kyphosis	67.6 (28.8)	[63.1, 72.2]	78.1 (23.9)	[70.0, 86.1]	0.027*
Post-operative kyphosis	42.3 (16.6)	[39.8, 44.9]	48.1 (14.9)	[43.1, 53.1]	0.042*
Raw change in kyphosis	− 26.1 (24.8)	[− 30.1, − 22.0]	− 29.5 (25.6)	[− 37.8, − 21.2]	0.463
Pre-operative pelvic obliquity	20.2 (12.8)	[18.1, 22.3]	23.8 (12.5)	[19.3, 28.4]	0.149
Post-operative pelvic obliquity	7.2 (6.4)	[6.2, 8.3]	8.6 (6.2)	[6.3, 11.0]	0.275
Percent change in pelvic obliquity	− 48.7 (84.4)	[− 63.5, − 33.9]	− 44.0 (64.7)	[− 68.7, − 19.4]	0.745

	*N*	%	N	%	*p*-value
Surgery type					
MCGR	81	47%	32	86%	< 0.001*
VEPTR/TGR	70	40%	2	5%	
Fusion	22	13%	3	8%	
Superior attachments					
Rib	64	42%	18	53%	0.500
Spine	82	54%	15	44%	
Both	5	3%	1	3%	
Inferior attachments					
Pelvis	61	40%	14	41%	> 0.999
Spine	30	20%	6	18%	
Spine and pelvis	59	39%	14	41%	
Rib and pelvis	1	1%	0	0%	

^*^Indicates significance, alpha = 0.05

**Fig. 3:**
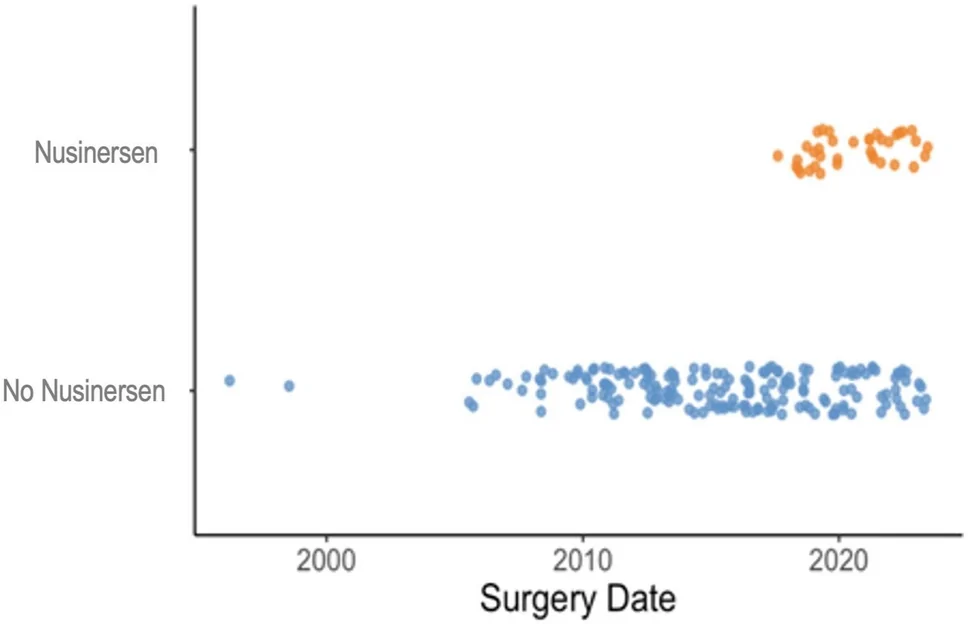
Scatter plot demonstrating the distribution of dates of surgery between the control and nusinersen groups

All patients in the nusinersen group had surgery after August 2017. 52% (n = 90) of patients in the control group had surgery prior to this date, and 48% underwent surgery after August 2017 (Fig. 3).

Patients treated with nusinersen were younger, with a mean age at surgery of 6.5 years (SD 2.0) compared to 7.6 years (SD 2.8) in the control group (p = 0.006). In terms of scoliosis curve characteristics, mean preoperative kyphosis was higher among patients with nusinersen use vs controls, 78° vs. 67° (*p* = 0.027). Postoperative thoracic kyphosis was greater in the nusinersen group compared to the control group, 48° vs 42° (*p* = 0.042). The mean preoperative major curve magnitude was also higher among patients with nusinersen use, 83° vs. 75° (*p* = 0.030). Postoperative major curve magnitude and percent correction of the major curve did not differ between the two groups. The mean change in kyphosis did not differ between the groups. The mean preoperative and postoperative pelvic obliquity and percent correction did not differ between the groups (Table 1).

Patients in both groups predominantly underwent surgery with growth-friendly instrumentation. In the control group, there was a higher proportion of vertical expandable prosthetic titanium rib/ traditional growth rods (VEPTR/TGR) use than the nusinersen group (40% vs. 5%, *p *< 0.001), while the nusinersen group was more commonly treated with magnetically controlled growing rods (MCGR) than the control group (86% vs. 47%, *p* < 0.001). Patients in the nusinersen group had longer instrumentation constructs, with a mean of 10.5 (SD 2.5) levels spanned compared to the control group of 9.5 (SD 2.1) levels (*p* = 0.033). There were no differences between groups regarding rates of fusion surgery or superior or inferior anchor use to the ribs, spine, or pelvis. (Table 1).

We performed a subanalysis of the 108 patients (71 control, 37 with nusinersen exposure) undergoing index surgery after 2017 in order to minimize the influence of changing surgical indications and practice patterns on the results. Similar to the main analysis, in the post–2017 groups, patients treated with nusinersen were younger at the time of surgery, with a mean age of 6.5 years (SD 2.0) compared to 7.8 years (SD 2.8) in the control group (*p* = 0.010). The mean preoperative major curve magnitude among patients with nusinersen use was 83° vs. 81° among controls, and this difference was not statistically significant (*p* = 0.563). Mean preoperative kyphosis among patients with nusinersen use was 78° vs controls at 76° (*p* = 0.680), and this difference was not statistically significant. In contrast to the main analysis, patients in both groups primarily underwent surgery with MCGR, and there was no difference between groups in the distribution of patients treated with growing constructs vs. fusion. Similar to the main analysis, patients in the nusinersen group had longer instrumentation constructs, with a mean of 10.5 (SD 2.5) levels spanned compared to the control group of 9.4 (SD 2.0) levels (*p* = 0.023) (Table 2).

**Table 2:** Demographics, surgical, and curve characteristics by nusinersen use (subanalysis including only patients treated surgically after 2017)

	No nusinersen use (*n* = 71)		Nusinersen use (*n* = 37)		
	Mean (SD)	95% CI	Mean (SD)	95% CI	*p*-value
Age (years)	7.8 (3.2)	[7.1, 8.6]	6.5 (2.0)	[5.9, 7.2]	0.010*
BMI	17.3 (4.2)	[16.2, 18.4]	16.2 (3.0)	[14.9, 17.4]	0.181
Instrumented levels	9.4 (2.0)	[8.9, 9.9]	10.5 (2.5)	[9.7, 11.3]	0.023*
Pre-operative cobb angle	80.9 (21.4)	[75.9, 86.0]	83.3 (19.5)	[76.8, 89.8]	0.563
Post-operative cobb angle	38.5 (14.8)	[35.0, 42.0]	45.3 (19.3)	[38.9, 51.7]	0.065
Percent change in cobb angle	− 51.7 (16.3)	[− 55.6, − 47.8]	− 46.3 (16.6)	[− 51.9, − 40.8]	0.114
Pre-operative kyphosis	75.8 (29.7)	[68.3, 83.3]	78.1 (23.9)	[70.0, 86.1]	0.680
Post-operative kyphosis	47.0 (17.0)	[42.9, 51.1]	48.1 (14.9)	[43.1, 53.1]	0.732
Raw change in kyphosis	− 29.2 (27.5)	[− 36.2, − 22.2]	− 29.5 (25.6)	[− 37.8, − 21.2]	0.959
Pre-operative pelvic obliquity	22.5 (13.4)	[19.1, 25.9]	23.8 (12.5)	[19.3, 28.4]	0.640
Post-operative pelvic obliquity	7.0 (6.2)	[5.5, 8.5]	8.6 (6.2)	[6.3, 11.0]	0.245
Percent change in pelvic obliquity	− 65.0 (32.6)	[− 73.5, − 56.5]	− 44.0 (64.7)	[− 68.7, − 19.4]	0.108

	N	%	N	%	*p*-value
Type					
MCGR	54	76%	32	86%	0.094
VEPTR/TGR	1	1%	2	5%	
Fusion	16	23%	3	8%	
Superior attachments					
Rib	12	22%	18	53%	0.002*
Spine	43	78%	15	44%	
Both	0	0%	1	3%	
Inferior attachments					
Pelvis	6	11%	14	41%	0.003*
Spine	9	16%	6	18%	
Spine and pelvis	40	73%	14	41%	
Rib and pelvis	0	0%	0	0%	

^*^Indicates significance, alpha = 0.05

Given that our cohort spans a group of patients with variable duration of preoperative exposure to nusinersen, and it is not established what duration of preoperative treatment would affect a change on curve characteristics, we performed sensitivity analyses based on duration of preoperative treatment. We performed two such analyses, one including only patients who had greater than 1 year (*n* = 23) of exposure to nusinersen preoperatively and another including only patients who had greater than 2 years (*n* = 16) of exposure to nusinersen preoperatively comparing both of these groups to the same set of 173 control patients. Similar to the main analyses, patients treated for at least 1 year were younger at the time of surgery than untreated patients (6.6 [SD 2.0] vs. 7.6 [SD 2.8], *p* = 0.034), had greater preoperative major curve magnitude (85° vs. 75°, *p* = 0.025), greater preoperative kyphosis (84° vs. 68°, p < 0.001), greater postoperative kyphosis (51° vs. 42°, *p* = 0.019), and underwent surgery with MCGR at a higher rate (96% vs. 47%, *p* < 0.001). In contrast to the previous analysis, postoperative major curve magnitude was significantly greater in the treated group (48° vs. 39°, *p* = 0.017) and the number of instrumented levels was not significantly different between groups (*p* = 0.244). Similar to the main analysis, patients treated for at least 2 years had greater preoperative kyphosis than untreated patients (88° vs. 68°, *p* = 0.001), greater postoperative kyphosis (54° vs. 42°, *p* = 0.023), and underwent surgery with MCGR at a higher rate (94% vs. 47%, *p* < 0.001). Though the means were similar between all treatment groups, age at surgery (6.7 [SD 2.8] vs. 7.6 [SD 2.0], *p* = 0.088) and major curve magnitude pre- (83.8° vs. 75.3°, *p* = 0.117) and post-operatively (47.6° vs. 38.5°, *p* = 0.054) were not significantly different between the 16 patients who had greater than 2 years of exposure to nusinersen preoperatively and controls.

## Discussion

In this cohort, we demonstrated that nusinersen use was associated with larger, more kyphotic curves and younger age at surgical intervention among patients with SMA undergoing initial surgical treatment for EOS. These patients were also more commonly treated with MCGR than VEPTR/TGR. These results highlight changes in the curve characteristics of patients with SMA undergoing surgery for scoliosis in the post-nusinersen era.

One potential confounder may be that, over the treatment period studied, surgeons are choosing to intervene later with growing instrumentation. In order to account for shifting indications, we performed a subgroup analysis of patients treated contemporaneously (after 2017). These patients had similar curve characteristics pre- and post-operatively regardless of nusinersen exposure. Notably, though, patients with nusinersen exposure arrived at those curve characteristics and had their index operation on average 1.3 years earlier than patients who were not exposed to nusinersen. This may suggest patients exposed to nusinersen have an earlier onset, more progressive scoliosis. However, this association does not prove a causal relationship between nusinersen treatment and worsening scoliosis in individual patients. The observed association could possibly reflect a changing patient population presenting to spine surgeons because of functional and longevity gains associated with DMA. Furthermore, the associations may also reflect shifting practice patterns among surgeons indicating intervention in scoliosis associated with SMA, as well as a closer supervision by multidisciplinary teams leading to different treatment approaches. Additionally, the longer mean instrumentation length in the nusinersen group (10.5 vs. 9.5 levels; *p* = 0.033) could be attributable to increased kyphosis seen in the treatment group which may have resulted in higher upper instrumented vertebra.

Multiple mechanisms may underlie the associations described. Given that nusinersen improves motor function and strength, increases lifespan, and decreases medical complications in patients with SMA, one explanation behind our findings is that nusinersen and other DMAs may be improving function among patients who previously, if untreated, may not have been considered surgical candidates. Before the DMA era, most SMA patients with surgically treated scoliosis were classified as Type II, since SMA Type I patients rarely survived long enough or achieved sufficient trunk control to develop scoliosis requiring surgical intervention, whereas Type II patients, who typically achieve the ability to sit but not walk, frequently developed progressive scoliosis and were the primary group considered for surgical management [26]. Now, with DMAs, patients who may have traditionally been classified as phenotypically SMA Type I have increased longevity and trunk strength sufficient to sit or stand [27]and thus have more to gain from surgical treatment of scoliosis. Expectations of gains in trunk strength and function may also encourage parents and therapists to position more severely affected children with poor trunk control upright more often, making flexible collapsing kyphosis more apparent. Whether this leads to structural deformity in time remains unclear. Our findings do not exclude the possibility that nusinersen or other DMAs improve the incidence of progressive scoliosis among patients who may have traditionally been classified as phenotypically SMA Type II. Though traditional SMA Type I patients now sit and have functional similarities to the pre-DMA Type 2 population, the skeletal manifestations of SMA may not be the same. Critically, a major limitation of this dataset is the lack of information regarding phenotypic (Type I, II, III) or genotypic (SMN2 copy number) classification of SMA disease severity. Without this data, we are unable to describe differential skeletal phenotypes across SMA subtypes of how this may interact with Nusinersen exposure to influence surgical outcomes.

While the effects of nusinersen use on motor function in patients with SMA have been well described, there is a need for more research on how this may impact scoliosis progression or curve characteristics. In an analysis of 29 patients with SMA type I, the authors found that 93.1% of patients had scoliosis and 69% had abnormal pelvic obliquity, and these findings had no significant association with patient age, total nusinersen administrations, or age at the first dose [24]. In another study of 7 patients with SMA type I, all patients who had received nusinersen within the first six months of their life still developed scoliosis and with Curve magnitudes between 18° and 60° by the end of their first year [20]. Additionally, the study demonstrated that scoliosis progression greater than a Curve magnitude of 40° was associated with decreased motor function. In a more recent retrospective cohort of 165 patients with SMA and a minimum 2-year follow-up, DMA use was associated with a decreased risk of scoliosis development [7]. In a study comparing the effect of nusinersen therapy across various types of SMA, treatment was associated with increased scoliosis progression in patients with SMA I yet decreased scoliosis progression in patients with SMA type II and III [21]. In contrast, a single center study from Brazil found that nusinersen treatment did not prevent scoliosis in 41 patients with types II and III SMA at 2-year follow-up [28]. These mixed results suggest that DMA may not be as impactful for the skeletal manifestations of SMA as it is on the strength and functional limitations. For example, patients traditionally classified as SMA type I who are treated with nusinersen at an early age may have function that matches or exceeds patients with SMA type 2 but the incidence and progressive nature of scoliosis in that setting may be similar or worse than that which is historically described in Type 2 SMA. Unfortunately, research into the associations between DMA and musculoskeletal manifestations of SMA is likely to be increasingly confounded as treatment paradigms rapidly shift. More DMAs have come on to the market since the introduction of nusinersen and patients are often switched between several therapies or using DMAs in combination. Furthermore, non-operative approaches to therapy and bracing are changing and may also influence the timing and rates of surgical intervention.

Our study had several limitations. We were able to obtain treatment history with regards to nusinersen usage, however, we do not have access to information about the usage of DMAs other than nusinersen which may confound any attempts to infer causal relationships from observed associations. Furthermore, because of our small sample size we elected to consider nusinersen exposure as a binary variable though we openly acknowledge that duration of exposure and age at initiation to nusinersen varied in our cohort. DMA treatment likely has different impacts on the functional status of patients depending upon baseline disease severity, age of treatment initiation, and duration of treatment [5, 29, 30]. In order to assess the impact of this decision on our results we performed sensitivity analyses based on duration of preoperative nusinersen exposure. In these subanalyses we included patients who had greater than 1 year or greater than 2 years of preoperative exposure to nusinersen and we found similar results to the main analysis indicating that patient's had more severe curvatures at younger ages. As the sample sizes decreased in the subanalyses, not all of the results reached statistical significance. We suspect that there is a continuous and cumulative impact of increasing duration of DMA exposure on scoliosis characteristics but in this data set we lack the sample size to consider nusinersen exposure as a continuous variable. Furthermore, as this was a multicenter study spanning decades of treatment, we could not systematically account for temporal variations in surgical indications or variations based on surgeon or practice setting. Finally, our analysis was limited to a cross-sectional assessment of the perioperative period and lacked long-term follow-up. Despite these limitations, this study represents the largest study investigating scoliosis curve characteristics and of patients with SMA undergoing surgical management of EOS spanning the introduction of DMA. Future research is needed to characterize how other variables such as DMA treatment duration, age of onset, copies of functioning SMN2 genes, and SMA disease severity may modulate effects of nusinersen on scoliosis.

## Conclusion

In summary, we found that nusinersen use was associated with younger age at surgery, increased curve magnitude, and kyphosis in patients with SMA undergoing surgical treatment for EOS. There are a number of possible mechanisms that could underlie the associations described here. We suspect that functional improvements among severely affected patients associated with DMA treatment are likely resulting in a shift in the genetic subtypes of patients indicated for surgery, and this shift may be resulting in a patient population that presents with more severe curves at younger ages. Surgical indications and treatment protocols continue to evolve and we also cannot exclude the possibility of nusinersen/DMA use resulting in earlier onset or more rapid progression of scoliosis. Though challenging, further prospective research with improved ability to control for confounders will be needed to establish causal relationships.

## Supplementary Information

Below is the link to the electronic supplementary material.

Supplementary file1 (DOCX 2864 KB)

## Data Availability

The data that support the findings of this study are available from the corresponding author, JSV, upon request.
