# Supplementary material for: A changing early onset scoliosis phenotype in spinal muscular atrophy: nusinersen treatment is associated with younger age, increased curve magnitude, and kyphosis at the time of index surgery
Source: Spine Deform. 2026 Sep 17;14(6):2297–305. doi: 10.1007/s43390-026-01359-1 (PMC13690840; doi:10.1007/s43390-026-01359-1)
Supplement: Supplementary file 1 [file 43390_2026_1359_MOESM1_ESM.docx]

**Supplementary Information**

**Table 3: Subanalysis, Nusinersen Use ≥ 1 Year**

|  | **No Nusinersen Use**  **(n = 173)** | | **Nusinersen Use ≥1 Year**  **(n = 23)** | |  |
| --- | --- | --- | --- | --- | --- |
|  | **Mean (SD)** | **95% CI** | **Mean (SD)** | **95% CI** | **p-value** |
| **Age (years)** | 7.6 (2.8) | [7.2, 8.1] | 6.6 (2.0) | [5.8, 7.5] | 0.034* |
| **BMI** | 16.8 (4.3) | [16.0, 17.5] | 16.2 (3.1) | [14.5, 17.9] | 0.498 |
| **Instrumented Levels** | 9.5 (2.1) | [9.2, 9.9] | 10.3 (2.8) | [9.1, 11.5] | 0.244 |
| **Pre-Operative Cobb Angle** | 75.3 (21.9) | [72.0, 78.6] | 84.9 (17.8) | [77.2, 92.6] | 0.025* |
| **Post-Operative Cobb Angle** | 38.5 (16.1) | [36.1, 40.9] | 48.0 (16.9) | [40.7, 55.3] | 0.017* |
| **Percent Change in Cobb Angle** | -47.9 (20.5) | [-51.0, -44.8] | -44.0 (13.0) | [-49.6, -38.3] | 0.213 |
| **Pre-Operative Kyphosis** | 67.6 (28.8) | [63.1, 72.2] | 83.9 (18.6) | [75.8, 91.9] | < 0.001* |
| **Post-Operative Kyphosis** | 42.3 (16.6) | [39.8, 44.9] | 51.2 (15.9) | [44.4, 58.1] | 0.019* |
| **Raw Change in Kyphosis** | -26.1 (24.8) | [-30.1, -22.0] | -32.7 (21.4) | [-41.9, -23.4] | 0.191 |
| **Pre-Operative Pelvic Obliquity** | 20.2 (12.8) | [18.1, 22.3] | 23.3 (12.4) | [17.3, 29.2] | 0.324 |
| **Post-Operative Pelvic Obliquity** | 7.2 (6.4) | [6.2, 8.3] | 8.5 (5.9) | [5.7, 11.4] | 0.383 |
| **Percent Change in Pelvic Obliquity** | -48.7 (84.4) | [-63.5, -33.9] | -40.0 (69.5) | [-73.5, -6.5] | 0.627 |
|  | **N** | **%** | **N** | **%** | **p-value** |
| **Surgery Type** |  |  |  |  |  |
| MCGR | 81 | 47% | 22 | 96% | < 0.001* |
| VEPTR/TGR | 70 | 40% | 0 | 0% |  |
| Fusion | 22 | 13% | 1 | 4% |  |
| **Superior Attachments** |  |  |  |  |  |
| Rib | 64 | 42% | 13 | 59% | 0.205 |
| Spine | 82 | 54% | 8 | 36% |  |
| Both | 5 | 3% | 1 | 5% |  |
| **Inferior Attachments** |  |  |  |  |  |
| Pelvis | 61 | 40% | 12 | 55% | 0.439 |
| Spine | 30 | 20% | 2 | 9% |  |
| Spine and Pelvis | 59 | 39% | 8 | 36% |  |
| Rib and Pelvis | 1 | 1% | 0 | 0% |  |

*Indicates significance, alpha = 0.05

**Table 4: Subanalysis, Nusinersen Use ≥ 2 Year**

|  | **No Nusinersen Use**  **(n = 173)** | | **Nusinersen Use >2 Year**  **(n = 16)** | |  |
| --- | --- | --- | --- | --- | --- |
|  | **Mean (SD)** | **95% CI** | **Mean (SD)** | **95% CI** | **p-value** |
| **Age (years)** | 7.6 (2.8) | [7.2, 8.1] | 6.7 (2.0) | [5.6, 7.7] | 0.088 |
| **BMI** | 16.8 (4.3) | [16.0, 17.5] | 16.6 (3.4) | [14.3, 18.9] | 0.867 |
| **Instrumented Levels** | 9.5 (2.1) | [9.2, 9.9] | 9.6 (2.0) | [8.5, 10.6] | 0.976 |
| **Pre-Operative Cobb Angle** | 75.3 (21.9) | [72.0, 78.6] | 83.8 (19.7) | [73.3, 94.3] | 0.117 |
| **Post-Operative Cobb Angle** | 38.5 (16.1) | [36.1, 40.9] | 47.6 (16.8) | [38.6, 56.5] | 0.054 |
| **Percent Change in Cobb Angle** | -47.9 (20.5) | [-51.0, -44.8] | -44.2 (9.7) | [-49.4, -39.1] | 0.211 |
| **Pre-Operative Kyphosis** | 67.6 (28.8) | [63.1, 72.2] | 87.6 (19.7) | [77.1, 98.0] | 0.001* |
| **Post-Operative Kyphosis** | 42.3 (16.6) | [39.8, 44.9] | 53.6 (17.2) | [44.4, 62.7] | 0.023* |
| **Raw Change in Kyphosis** | -26.1 (24.8) | [-30.1, -22.0] | -34.0 (23.9) | [-46.7, -21.3] | 0.225 |
| **Pre-Operative Pelvic Obliquity** | 20.2 (12.8) | [18.1, 22.3] | 19.4 (13.2) | [11.1, 27.8] | 0.846 |
| **Post-Operative Pelvic Obliquity** | 7.2 (6.4) | [6.2, 8.3] | 9.0 (5.7) | [5.4, 12.6] | 0.324 |
| **Percent Change in Pelvic Obliquity** | -48.7 (84.4) | [-63.5, -33.9] | -21.3 (80.1) | [-72.2, 29.6] | 0.280 |
|  | **N** | **%** | **N** | **%** | **p-value** |
| **Surgery Type** |  |  |  |  |  |
| MCGR | 81 | 47% | 15 | 94% | < 0.001* |
| VEPTR/TGR | 70 | 40% | 0 | 0% |  |
| Fusion | 22 | 13% | 1 | 6% |  |
| **Superior Attachments** |  |  |  |  |  |
| Rib | 64 | 42% | 8 | 53% | 0.332 |
| Spine | 82 | 54% | 6 | 40% |  |
| Both | 5 | 3% | 1 | 7% |  |
| **Inferior Attachments** |  |  |  |  |  |
| Pelvis | 61 | 40% | 10 | 67% | 0.250 |
| Spine | 30 | 20% | 1 | 7% |  |
| Spine and Pelvis | 59 | 39% | 4 | 27% |  |
| Rib and Pelvis | 1 | 1% | 0 | 0% |  |

*Indicates significance, alpha = 0.05
